# Using the Cleaner XT Device for Pharmacomechanical Thrombolysis of Arteriovenous Fistulas and Grafts

**DOI:** 10.7759/cureus.90571

**Published:** 2025-08-20

**Authors:** Jee Hyuk Byun, Preethi Jagannath, Junaid Raja

**Affiliations:** 1 Department of Radiology, University of Alabama at Birmingham, Birmingham, USA

**Keywords:** arteriovenous fistula, arteriovenous graft, cleaner xt, hemodialysis, rotational thrombectomy

## Abstract

Purpose: The Cleaner XT^TM^ (Argon Medical Device, Plano, TX) rotational thrombectomy (RT) device is an emerging treatment option for arteriovenous fistula (AVF) and graft revascularization. This study aims to report the procedural details, safety, efficacy, and patency of AVFs and grafts for endovascular salvage procedures conducted with the Cleaner XT^TM^ device.

Methods: All patients who underwent RT with the Cleaner XT^TM^ RT System for endovascular salvage of AVFs and grafts from August 2016 to July 2022 at a single tertiary hospital were included. Study population, procedure, lesion characteristics, and the patency of AVFs and grafts after endovascular thrombectomy were reported.

Results: Patency after endovascular salvage with Cleaner XT^TM^ for all 13 patients was 84.6% (11/13) at one-month follow-up and 53.8% (7/13) for all follow-ups until 12 months.

Conclusion: This study shows that endovascular access salvage of AVF and graft with Cleaner XT^TM^ is safe and demonstrates an excellent clinical and technical success rate.

## Introduction

As defined by the National Kidney Foundation's Dialysis Outcomes Quality Initiative Guidelines (KDOQI), arteriovenous fistulas (AVFs) have been considered the vascular access of choice for patients requiring hemodialysis (HD) due to end-stage renal disease [1,2]. For patients who are not surgical candidates for AVF creation, arteriovenous grafts (AVGs) can be used as an alternative vascular access [3].

Both HD accesses are prone to thrombosis due to stenosis, and failure to maintain vascular patency can result in thrombus solidification and loss of access [4]. Various techniques, including catheter-directed thrombolysis, percutaneous mechanical thrombectomy, and pharmacomechanical thrombolysis, have been established as effective endovascular salvage strategies [5]. Among these, pharmacomechanical thrombolysis is widely utilized in vascular access procedures due to its comparable efficacy to surgical thrombectomy in treating AVGs and AVFs, as well as its proven success in managing deep vein thrombosis (DVT) [6,7].

The Cleaner XT^TM^ (Argon Medical Device, Plano, TX) rotational thrombectomy (RT) device has been shown to be an effective pharmacomechanical thrombolysis device in the revascularization of dialysis accesses [8,9]. The Cleaner XT^TM^ device is recognized for its time-efficiency and clinical effectiveness in treating thrombosed vessels, with its benefits well-documented in studies involving DVT [10,11]. Recent studies have also reported the use of the CleanerXT^TM^ device for treating clotted AVGs and AVFs, highlighting its effectiveness in mechanical thrombolysis [8,9].

This study presents our experience with the CleanerXT^TM^ RT device in 13 consecutive patients undergoing endovascular salvage of dialysis access. We focus on procedural methodology, safety, efficacy, and patency outcomes in a U.S.-based cohort of AVG and AVF patients.

## Materials and methods

Patient cohort

This retrospective cohort analysis utilized data from a single U.S. tertiary-care academic hospital. All patients (n = 13) who underwent RT with the Cleaner XT^TM^ RT System due to a thrombosed dialysis circuit for endovascular salvage of AVF or AVG from August 2016 to July 2022 were included in the population.

The Cleaner XT^TM^ device was used for the endovascular salvage of three AVF and 10 AVG patients. All procedures were performed at the Interventional Radiology and Interventional Nephrology suite under local anesthesia. All participants received an explanation of the potential benefits and risks of the salvage procedure using the Cleaner XT^TM^ device before giving informed consent. The technical and clinical success rates were both 100% for all procedures.

All procedures performed in the study involving human participants were in accordance with the ethical standards of the institutional and/or national research committee and with the 1964 Helsinki declaration [12] and its later amendments or comparable ethical standards, with approval from the local institutional review board and waiver of consent.

Devices

The Cleaner XT^TM^ RT System is a battery-operated, catheter-based percutaneous device. It spins at approximately 4,000 to 5,000 RPM to break down clots in native vessel fistulae and synthetic vascular access grafts. The device consists of a rotator drive unit attached to a radio-opaque wire. The flexible “S”-shaped guide wire spins inside the vessel to gently macerate the clot, which is then aspirated through an introducer sheath.

Device selection

Device selection was at the discretion of the treating interventionalist, but in practice, Cleaner XT^TM^ was typically chosen for thrombosed accesses suspected to contain chronic or wall-adherent thrombus, aneurysmal segments, or diffuse narrowing where direct wall-contact maceration was deemed advantageous. It was generally avoided in grafts or fistulas with sharply angulated segments or in cases where thrombus extended close to the arterial anastomosis due to the higher risk of embolization. In such situations, alternative mechanical or pharmacomechanical approaches were preferred.

Definitions

Technical success was defined as less than 30% of stenosis after the completion of the procedure. Clinical success was defined as resumption of HD for at least one session. The conditions for procedural success were met if both technical and clinical success were achieved. Secondary patency was defined as the time during which the vascular access was patent until it required the next endovascular dialysis salvage. The Society of Interventional Radiology (SIR) Contracted Accordion Complication Classification was used to classify procedural complications based on severity.

Procedure techniques

**Figure 1:**
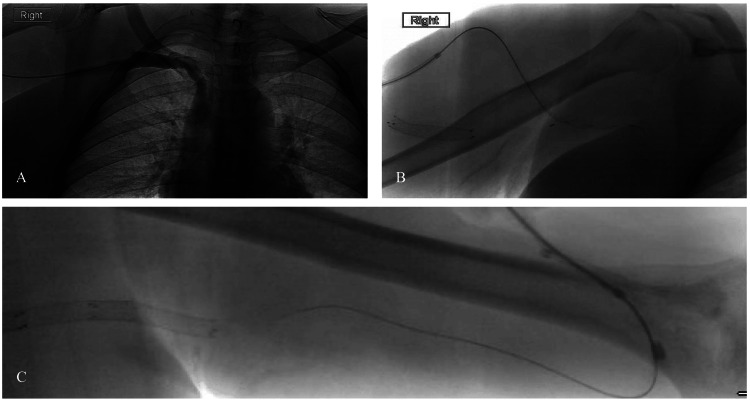
(A) Central run showing the level of thrombosis. (B) Antegrade application of the Cleaner XT device. (C) Retrograde application and pullback with the Fogarty balloon

**Figure 2:**
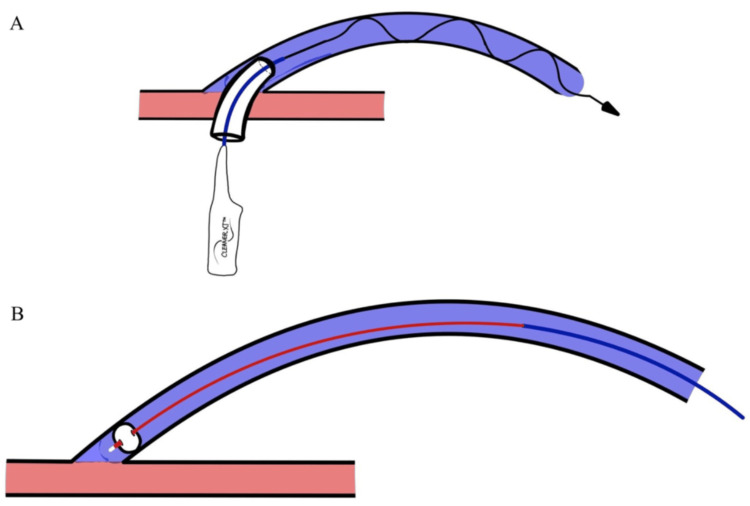
(A) Antegrade application of the Cleaner XT device. (B) Retrograde application and pullback with the Fogarty balloon Source: Reused with permission from the original publisher, Sage Publications (The Journal of Vascular Access) [13]

Figure 1 illustrates real-time procedural images of thrombectomy for AVGs and AVGs using the Cleaner XT^TM^ RT and balloon maceration (BM). Figure 2 provides a detailed depiction of the antegrade application of the Cleaner XT^TM^ device and the retrograde application with pullback using the Fogarty balloon.

Antegrade access was typically established first using a 21-gauge needle and a microintroducer set, which facilitated the transition to a 7-Fr 5-cm sheath. A 0.035-inch wire and a 5-Fr Kumpe catheter were then advanced centrally, followed by pullback angiography to delineate the extent of the thrombosis. If administered, tissue plasminogen activator (tPA) and heparin were delivered through the catheter at this stage. Before inserting the Cleaner XT^TM^ device, the original antegrade guide wires were removed. Thrombolysis was performed under ultrasound and fluoroscopic visualization using the battery-operated rotational Cleaner XT^TM^ device (Figures 1B, 2A).

Retrograde access was obtained with a 21-gauge needle and a microintroducer catheter. A Kumpe catheter and a 0.035-inch wire were used to gain retrograde access into the brachial artery. A Fogarty balloon was then advanced into the artery, inflated, and pulled back across the arterial anastomosis to dislodge the clot at the arteriovenous anastomosis (Figures 1C, 2B). The plug at the arterial anastomosis was then dislodged into the tPA-infused bloodstream. The Cleaner XT^TM^ device was exclusively used from the antegrade access and was never employed in retrograde access, as advancing it closer to the arterial anastomosis posed a risk of clot displacement into the arterial circulation. Fogarty balloons were the preferred device for retrograde removal of clots for the arterial anastomosis.

Following thrombectomy with the Cleaner XT^TM^ device, balloon angioplasty was performed in selected cases to expand stenotic regions. A follow-up angiogram of the dialysis circuit, obtained from the brachial artery, confirmed a widely patent circuit without significant thrombosis or stenosis. The sheaths were then removed, and hemostasis was achieved with manual pressure.

The decision to use the Cleaner XT^TM^ device was based on operator preference. RT with this device was typically chosen for cases suspected of chronic clot formation or aneurysm. While it was not used in grafts or fistulas with excessively sharp angles, it remained an option for cases with diffuse narrowing and stenotic segments. None of the cases required balloon angioplasty before Cleaner XT^TM^ use due to these narrowing patterns. To minimize complications, all procedures were performed under real-time visualization, ensuring that excessive forward pressure was avoided.

## Results

Baseline characteristics of the study population

A total of 13 patients with thrombosed AVGs or AVFs underwent endovascular HD salvage using the Cleaner XT^TM^ device between August 2016 and July 2022. The baseline characteristics of the study population are presented in Table 1.

**Table 1:** Baseline characteristics for study population SD: standard deviation; BMI: body mass index

Demographics	Values, number (%), mean (±SD), or median (range)
Age (years)	53.2 (±9.9)
Gender	
Male	3 (23.1%)
Female	10 (76.9%)
BMI	31.0 (±8.8)
Comorbidities	
Hypertension	13 (100%)
Diabetes mellitus	12 (92.3%)
Hyperlipidemia	11 (84.6%)
Ischemic heart disease	8 (61.5%)
Cerebrovascular disease	5 (38.5%)
Active smoker	1 (7.7%)
History of anticoagulant use	2 (15.4%)
Underwent prior declot	11 (84.6%)
Days from prior declot	32 (2-516)
Underwent repeat declot	6 (46.2%)
Days to repeat declot	51 (21-85)

The mean age of the patients who underwent salvage thrombectomy with the Cleaner XT^TM^ device was 53.2 ± 9.9 years. Of the 13 patients, 76.9% (10/13) were female patients, with an average body mass index of 31.0 ± 8.8. Among all comorbidities, hypertension was the most prevalent, affecting 100% of the patients. Additionally, 15.4% (2/13) had a history of anticoagulant use. Prior endovascular salvage thrombectomy had been performed in 84.6% (11/13) of the patients, with a median interval of 32 days since the last procedure. Repeat endovascular salvage thrombectomy was required in 46.2% (6/13) of patients, with a median interval of 51 days until the next procedure.

Procedure and lesion characteristics

Table 2 presents the procedural and lesion characteristics. Among the thrombosed dialysis accesses that underwent endovascular salvage, 77.0% (10/13) were AVGs, while 23.0% (3/13) were AVFs. The distribution of vascular access sites was as follows: 30.8% (4/13) in the right upper arm, 38.5% (5/13) in the left upper arm, 23.1% (3/13) in the left lower arm, and 7.7% (1/13) in the left lower extremity.

**Table 2:** Procedure and lesion characteristics AVG: arteriovenous graft; AVF: arteriovenous fistula; tPA: tissue plasminogen activator

Demographics	Values, number (%) or median (range)
Vascular access type	
AVG	10 (77.0%)
AVF	3 (23.0%)
Vascular access location	
Right upper arm	4 (30.8%)
Left upper arm	5 (38.5%)
Left lower arm	3 (23.1%)
Left lower extremity	1 (7.7%)
Balloon location	
Inflow	2 (15.4%)
Outflow	3 (23.1%)
Both inflow and outflow	8 (61.5%)
Largest size balloon used	
6 mm	4 (30.8%)
7 mm	1 (7.7%)
8 mm	8 (61.5%)
Stent used during procedure	
Yes (size: 9 mm × 6 cm)	2 (15.4%)
No	11 (84.6%)
tPA used during the procedure	
Yes	8 (61.5%)
No	5 (38.5%)
tPA volume (mg)	3.25 (2-6)
Complications	0 (0.0%)

Balloon placement during the procedure was recorded in 15.4% (2/13) of cases for inflow, 23.1% (3/13) for outflow, and 61.5% (8/13) for both inflow and outflow. The largest balloon size used was 6 mm in 30.8% (4/13) of patients, 7 mm in 7.7% (1/13), and 8 mm in 61.5% (8/13). Stenting was required in 15.4% (2/13) of patients using a 9 mm × 6 cm stent. tPA was administered in 61.5% (8/13) of cases, with a median dose of 3.25 mg. No procedure-related complications were reported, as classified by the SIR Contracted Accordion Complication Classification [14].

Secondary patency rates after endovascular salvage with the Cleaner XT^TM^ device

**Figure 3:**
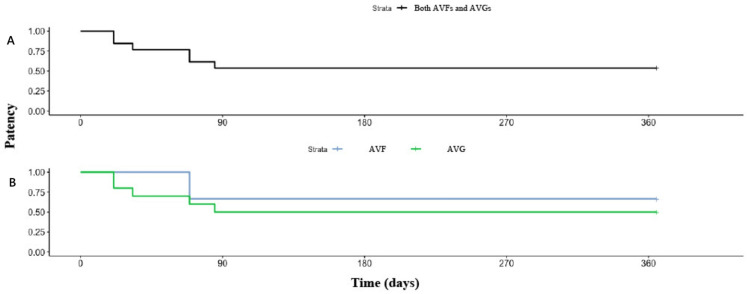
Patency rates following endovascular salvage with Cleaner XT for (A) combined AVG and AVF patients, and (B) AVG and AVF patients analyzed separately AVG: arteriovenous graft; AVF: arteriovenous fistula

Kaplan-Meier plots in Figure 3 illustrate secondary patency rates following endovascular salvage with the Cleaner XT^TM^ device. At one-month follow-up, secondary patency was 84.6% (11/13) for all patients and 53.8% (7/13) for all follow-ups up to 12 months. For AVGs, secondary patency was 100% (3/3) at one month and 66.7% (2/3) for all follow-ups up to 12 months. For AVGs, secondary patency was 80.0% (8/10) at one month and 50.0% (5/10) at 12 months.

Technical and clinical success rates

The KDOQI Vascular Access Guidelines [2] recommends that reasonable goals for graft patency would include a clinical success rate of >85% and a three-month primary patency rate of 40%. In our study, the overall clinical and technical success rates were 100%, and the three-month primary patency rate was 53.8% (7/13), which meets the KDOQI guidelines.

## Discussion

Pharmacomechanical thrombectomy offers distinct advantages over other thrombectomy methods by integrating mechanical thrombectomy with catheter-directed thrombolysis. This dual approach enhances the management of extensive thrombosis by combining mechanical clot disruption with enzymatic dissolution, optimizing efficacy and clot resolution. Several different pharmacomechanical thrombectomy devices with different thrombectomy mechanisms have been used and reported in the literature for clotted grafts and fistulas.

Currently, pharmacomechanical thrombectomy devices can generally be categorized into two types: rotational and rheolytic thrombectomy devices. Cleaner XT^TM^ falls under the category of rotational devices [15]. Rotational devices offer advantages over rheolytic devices due to their direct wall contact, allowing them to macerate clots from the vessel lumen. In contrast, rheolytic devices rely solely on high-pressure saline jets to break down clots without direct wall interaction [15]. Rheolytic devices can be potentially more prone to leaving behind residual thrombus adherent to the wall because they do not make direct contact with graft or fistula walls.

**Figure 4:**
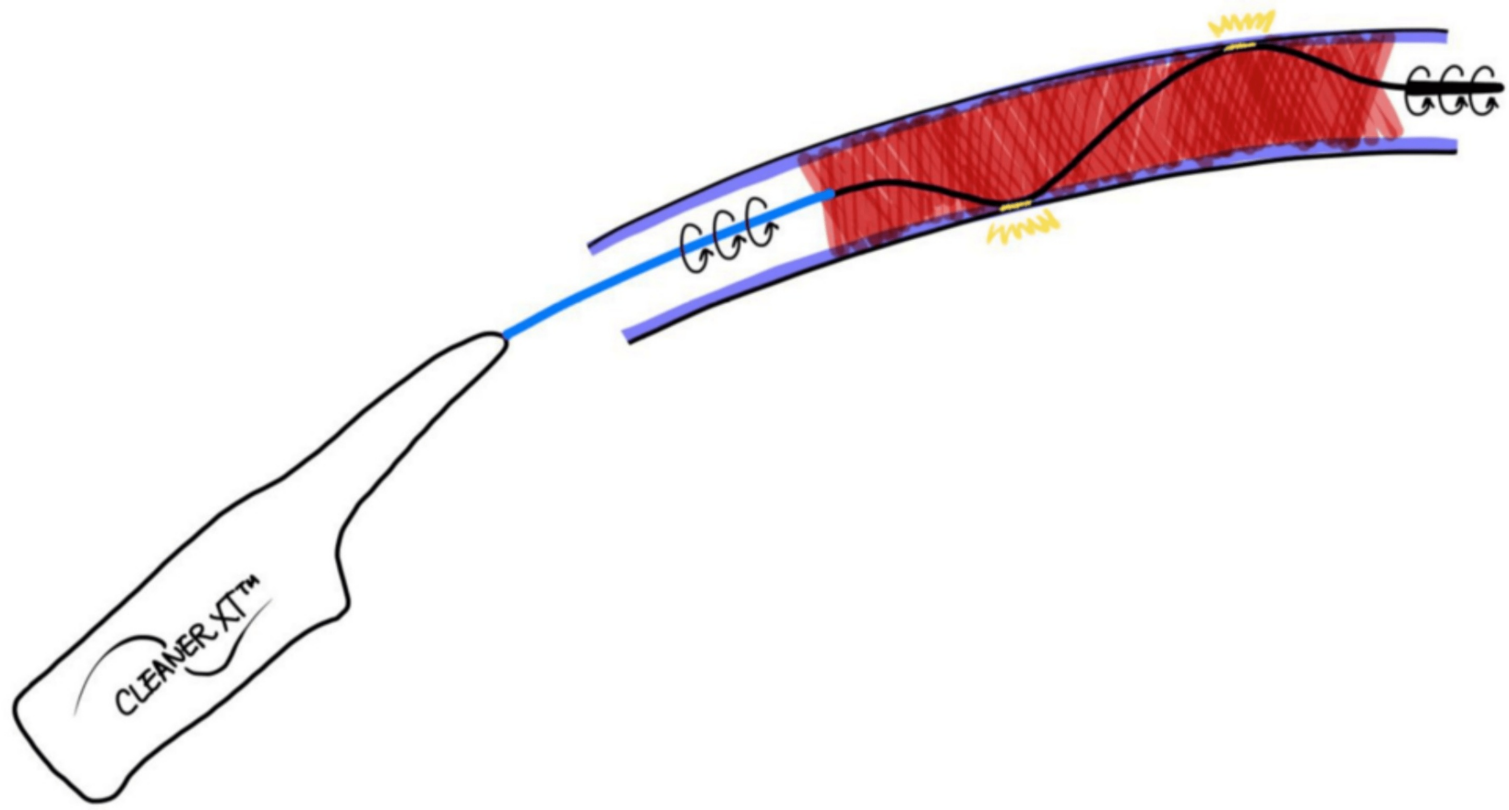
Cleaner XT as a wall-contact device Source: Reused with permission from the original publisher, Sage Publications (The Journal of Vascular Access) [13]

A study [16] including 275 thrombectomy procedures to compare the patency rate of AVFs after percutaneous thrombectomy using rheolytic and RT devices showed that AngioJet (Boston Scientific Corporation, Maple Grove, MN) rheolytic thrombectomy had a lower postintervention secondary patency rate at one year compared to Arrow-Trerotola RT (Teleflex, Wayne, PA; p = 0.01). The difference in the patency rate may be attributed to rheolytic devices, depending on the sucking of clots, potentially being less effective for older, more organized, and wall-adherent clots. Similar to the Arrow-Trerotola rotational device as a wall-contact rotational device, the Cleaner XT^TM^ thrombectomy system macerates and strips clots off graft or fistula walls (Figure 4). The device can be particularly beneficial for AVGs and AVFs with aneurysmal changes, especially when manual compression of the aneurysmal segment is applied during the procedure [8,9].

Another study [13] involving 27 patients who underwent either RT with the Cleaner XT^TM^ device (13 patients; 48.15%) or BM (14 patients; 51.85%) found that the median time to the next dialysis salvage after the initial procedure was longer for Cleaner XT^TM^. Specifically, the median time was 51 days for Cleaner XT^TM^ compared to 43.5 days for BM (W + 9, critical value 1). The inherent design of Cleaner XT^TM^ as a wall-contact device may have contributed to the prolonged graft patency compared to BM, which lacks the mechanical disruption of wall-adherent clots.

Compared to the studies by Huan et al. [9] and Bong et al. [8], which also examined the efficacy and safety of the Cleaner XT^TM^ for treating thrombosed AVGs and AVFs, our study utilized the Cleaner XT^TM^ strictly in an antegrade approach to minimize the risk of embolization. Despite this limitation, the Cleaner XT^TM^ achieved a 100% clinical and technical success rate with a 0% complication rate, in contrast to complication rates of 23.5% in Huan et al. [9] and 5.9% in Bong et al. [8] (Table 3). Additionally, the patency rates in our study at 1, 3, 6, and 12 months were 85%, 54%, 54%, and 54%, respectively, compared to 89%, 80%, 68%, and 59% in Bong et al. [8], and 65%, 47%, 47%, and 47% in Huan et al. [9] (Table 3). These findings suggest that utilizing the Cleaner XT^TM^ in an antegrade-only approach does not compromise effective clot removal while reducing the risk of embolization. The antegrade-only technique may offer a safer alternative for patients at risk of embolization without sacrificing thrombectomy efficacy.

**Table 3:** Comparison of patency rates for AVGs and AVFs after rotational thrombectomy with Cleaner XT for our study and two other studies AVG: arteriovenous graft; AVF: arteriovenous fistula

Studies	Patency rate (%)			
	1 month	3 months	6 months	12 months
Byun et al. [13]	85	54	54	54
Bong et al. [8]	89	80	68	56
Huan et al. [9]	65	47	47	47

Compared to our study and the study by Bong et al. [8], both of which used the Cleaner XT^TM^ as the primary thrombectomy device in all cases, Huan et al. [9] utilized Cleaner XT^TM^ only as a rescue tool for removing residual thrombus after thrombolytic agents and BM. This limited use of Cleaner XT^TM^ may have contributed to the steepest decline in patency rates, with a one-month patency of 65%, compared to 85% in our study and 89% in Bong et al. [8] (Table 3). Additionally, the follow-up patency rates at 3, 6, 9, and 12 months were the lowest in Huan et al. [9], at 47%, 47%, and 47%, respectively, compared to 54%, 54%, and 54% in our study and 80%, 68%, and 59% in Bong et al. [8] (Table 3). The restricted use of Cleaner XT^TM^ as a rescue device may have played a role in these discrepancies in patency outcomes.

Among the three studies, Bong et al. [8] reported the highest follow-up patency rates compared to our study and Huan et al. [9] (Table 3). This superior patency rate may be attributed to the combined use of both Cleaner XT^TM^ RT and balloon angioplasty in all cases. In contrast, our study utilized balloon angioplasty selectively, only in cases where it was deemed necessary after thrombectomy with the Cleaner XT^TM^ device. In Huan et al. [9], balloon angioplasty was performed in all cases, but Cleaner XT^TM^ was used only as a rescue tool for residual thrombus removal. The routine combination of Cleaner XT^TM^ and balloon angioplasty in Bong et al. [8] may have played a key role in achieving higher patency rates.

Limitations

This investigation is constrained primarily by its retrospective, single-center design and the small cohort size (n = 13), which limits statistical power and generalizability. Because device selection was left to operator discretion, there is an inherent risk of selection bias toward lesions perceived as amenable to RT, and no control group treated with alternative pharmacomechanical or surgical techniques was available for comparison. Procedural heterogeneity, such as adjunctive use of tPA, balloon angioplasty, and variable stent deployment, may have confounded the patency outcomes, and the absence of core-laboratory adjudication introduces the possibility of measurement bias. Follow-up was limited to a maximum of 12 months, with attrition over time, so late failures and long-term complications may be underreported. Finally, as a single-institution experience, the results reflect the expertise and protocols of one tertiary center and may not be reproducible in settings with different resources or operator skill levels.

Reproducibility across centers may also be influenced by the absence of a standardized, blinded review process for angiographic findings. In our study, imaging interpretation was performed by the operating interventionalist, and there was no independent review by multiple observers or adjudication by a core laboratory. While this reflects common real-world practice in many centers, it introduces the potential for subjectivity in assessing lesion characteristics, procedural endpoints, and residual thrombus. Future multicenter studies incorporating standardized imaging protocols, blinded multireader assessments, or centralized core-lab adjudication could reduce variability and enhance reproducibility of these findings across institutions.

Although device selection was determined by operator preference, the choice was informed by consistent practical considerations, such as clot chronicity, wall adherence, aneurysmal degeneration, and vessel geometry, which may limit generalizability. Because these criteria were not formally protocolized, subtle differences in operator judgment could have influenced outcomes. Future studies should define explicit selection algorithms to minimize subjective variability. Because device use was determined by operator preference, there is potential for selection bias toward cases perceived to have more favorable anatomy or thrombus characteristics for RT. This may partly explain the high technical and clinical success rates observed, and the findings should be interpreted with this in mind.

Finally, as this represents a small, single-center experience, the results should be considered hypothesis-generating. Larger scale retrospective or prospective studies are required to confirm the reproducibility, safety profile, and long-term patency outcomes associated with the Cleaner XT^TM^ device in dialysis access salvage.

## Conclusions

Our experience demonstrates that the Cleaner XT^TM^ RT device offers a technically straightforward and clinically effective option for salvaging thrombosed dialysis accesses, achieving 100% technical and clinical success with no device-related complications and acceptable secondary patency through 12 months. These findings contribute to the growing body of evidence that direct wall-contact rotational systems can achieve rapid declotting while minimizing the risk of residual mural thrombus and distal embolization, although interpretation is limited by the small sample size and other limitations mentioned. Although the initial results meet, and in some metrics exceed KDOQI performance benchmarks, they should be interpreted within the context of the study's methodological limitations. Future prospective, multicenter trials with larger, stratified cohorts and randomized comparisons against rheolytic and purely pharmacologic approaches are warranted to confirm durability, clarify cost-effectiveness, and identify patient or lesion characteristics that predict superior outcomes with Cleaner XT^TM^. Incorporating standardized imaging endpoints, blinded outcome adjudication, and health-related quality-of-life measures would further refine the evidence base. Until such data are available, our results support the selective use of Cleaner XT^TM^ as a safe and efficient first-line tool, particularly in accesses with chronic, wall-adherent thrombus or aneurysmal degeneration, while underlining the importance of tailored periprocedural anticoagulation, vigilant surveillance, and prompt reintervention to sustain long-term access patency in the HD population.
